# The phage for PPLA age: effective method for *Salmonella*-free poultry feed

**DOI:** 10.1128/spectrum.02877-24

**Published:** 2026-02-12

**Authors:** Elżbieta Fornal, Anna Pękala, Marcela Łaszkiewicz, Monika Sakosik, Weronika Muszyńska, Michał Psujek, Justyna Matczak, Małgorzata Stańczyk, Nicole L. Holcombe, Cheryl E. Schaeffer, Ewelina A. Wójcik, Jarosław Dastych

**Affiliations:** 1 Proteon Pharmaceuticals S.A. 724621 , Łódź, Poland; 2 Anitox Corporation 732524 , Lawrenceville, Georgia, USA; 3 The Institute of Medical Biology of the Polish Academy of Sciences, Łódź, Poland; The University of Tennessee Knoxville, Knoxville, Tennessee, USA

**Keywords:** bacteriophages, PPLA, *Salmonella*, poultry, feed

## Abstract

*Salmonella* is one of the most significant foodborne pathogens worldwide. Among the various sources of human *Salmonella* infections, poultry and poultry products are the most common. One of the primary routes of *Salmonella* transmission into poultry is through contaminated feed. In the present study, we evaluated the potential of bacteriophage cocktail BAFASAL applied via post-pellet liquid application (PPLA), at two concentrations—D1 – 1 × 10⁴ PFU/g and D2 – 5 × 10⁴ PFU/g—to prevent and reduce *Salmonella* contamination in poultry feed. In an *in vitro* experiment, where feed was artificially contaminated with *Salmonella* under laboratory conditions, both doses reduced *Salmonella* spp. counts by at least three logs compared to the untreated control feed. An *in vivo* challenge study in broiler chickens further demonstrated the protective effect of BAFASAL delivered via PPLA, confirming its effectiveness in a live animal model. After 3 days of feeding *Salmonella*-contaminated feed, no *Salmonella* was detected in crop samples from birds receiving BAFASAL/PPLA-treated feed. In contrast, *Salmonella* was detected in 7 out of 10 crop samples from control birds receiving untreated feed. Furthermore, analysis of cecal contents on days 18 and 35 revealed a statistically significant reduction in *Salmonella* colonization by more than four logs in the groups fed with feed protected by PPLA application of BAFASAL compared to the control group.

**IMPORTANCE:**

Bacteriophages, representing the most common biological entity on Earth, are viruses that infect bacteria. In recent years, there has been a revived interest in their practical application because of the antimicrobial resistance crisis. One growing commercial application of bacteriophages is animal farming and food processing to reduce the risk of pathogens passing through the food chain into humans. The use of bacteriophages may help reduce the risk of contamination of the food chain with bacteria resistant to antibiotics, among which *Salmonella* is recognized as a high concern pathogen for public health. Therefore, the use of bacteriophage cocktail BAFASAL to prevent *Salmonella* contamination in poultry feed is a promising and safe alternative to currently used antimicrobial agents and may be considered a solution in ensuring feed safety. Bacteriophage delivery to feed as post-pellet liquid application (PPLA) allows for large-scale usage in industrial poultry farms.

## INTRODUCTION

The poultry industry has experienced a rapid intensification of production since the early 20th century, across the broiler meat, laying hen, breeding stock, and turkey sectors. Rapid intensification was partly achieved by advances in genetics and in farm management, but was also supported by advances in nutrition, such as feed manufacturing and delivery of nutrients to birds (1). Recently, one of the main challenges of the poultry industry is feed production, where the selection of sustainable solutions for hygienic feed production is becoming the key as the antibiotic-free/no antibiotics ever (ABF/NAE) trend becomes more widespread and stronger. Previously, ABF production considered only some of the markets, such as the European Union (EU) and US, that introduced a ban on the usage of antibiotics as growth promoters, which has now become a global trend and a regular topic of discussion among animal health professionals in all regions of the world (2). Control of feed-related microbiological contamination, including contamination with *Salmonella*, without usage of antibiotics has become an important issue for animal and, as a consequence, human health (3). In the case of *Salmonella*, infected birds and their fecal material are the major sources of water and food contamination, not only directly contaminating the food chain but also spreading these pathogenic bacteria to the environment and, subsequently, indirectly increasing the risk of contamination of the food supply chain (4). Salmonellosis is one of the most frequently reported foodborne gastrointestinal infections in humans and a major cause of foodborne outbreaks worldwide; thus, it is a top food safety concern (5). Preventing salmonellosis and reducing *Salmonella* transmission through food and other routes requires a comprehensive approach at farm, production, distribution, and consumer levels (3, 6). *Salmonella* can infect poultry via a variety of sources, including contaminated feed, water, litter, pests, and the surrounding environment (7). In 2023, the overall EU-level occurrence of *Salmonella*-positive sampling units in any “animal and vegetable-derived feed” was 0.48% (*N* = 59337); in compound feed, the prevalence of *Salmonella*-positive units was 0.54% for samples from cattle feed (*N* = 1120), 0.38% for samples from poultry feed (*N* = 9267), 0.43% for samples from pig feed (*N* = 2824), and 1.0% for samples from pet food (*N* = 3163) (8).

Feed contains grain, milling by-products, animal by-products, vitamins, mineral supplements, fats, and oils (https://www.fda.gov/animal-veterinary/animal-foods-feeds/animal-food-ingredients), whereby animal by-products, grains, and sometimes fats and oils can be sources of *Salmonella* to varying degrees (7, 9). In addition, there are many routes of feed contamination that might occur during the storage and transportation of feed, the production process, or when hygiene conditions are inadequate (10). Controlling *Salmonella* in feed requires a wide range of approaches at different points where contamination occurs in the feed chain, including identification of risk factors for contamination and evaluation of the efficacy of interventions (3).

To ensure the manufacture of safe feed, comprehensive control strategies are implemented, among which physical and chemical intervention methods are the most common (10, 11). Prevention, such as implementing a feed mill biosecurity plan, thermal processing (pelleting and/or extrusion), and irradiation (gamma, UV, or electron beam) is used as a physical intervention (11). Furthermore, chemical interventions are also used to inhibit microbial growth and can include the use of various additives such as organic acids, formaldehyde, medium-chain fatty acids, and essential oils (11). These methods must be carefully managed to ensure safe production, as formaldehyde, while efficacious in decontaminating feed, is a hazardous material (12). In comparison, organic acids are not as hazardous but require higher inclusion rates (1%) and application for several days for equivalent efficacy (13). However, their use does not completely eliminate the risk of contamination by *Salmonella* in feed (11). It is worth noting that *Salmonella* contamination of feed, if not controlled, may also contribute to the transfer of antimicrobial-resistant bacteria into the food chain (14). Antimicrobial resistance is considered by the World Health Organization (WHO) as one of the top global public health and development threats. *Salmonella* is among the microorganisms in which resistant serotypes have emerged, affecting the food chain (https://www.who.int/news-room/fact-sheets/detail/antimicrobial-resistance; 15). In the 2024 edition of the WHO Bacterial Priority Pathogen List, *Salmonella* was classified as a high-priority pathogen, reflecting its increasing resistance to treatment and the significant infection burden it causes (https://www.who.int/publications/i/item/9789240093461). Furthermore, data published in the latest EFSA and ECDC report (2025) indicate high levels of resistance to ampicillin, sulfonamides, and tetracyclines in *Salmonella* spp. isolates from humans in 2023. Resistance levels ranged from moderate to very high in isolates from food-producing animals, except for laying hens, where resistance was low.

Bacteriophages (phages, BPs) represent a proven and already-implemented solution for controlling *Salmonella* contamination and reducing antibiotic usage. In recent years, several bacteriophage products targeting *Salmonella* spp., *Listeria monocytogenes*, *Shigella* spp., *Escherichia coli* O157:H7, and *Campylobacter* spp. have been granted Generally Recognized as Safe (GRAS) status by the U.S. Food and Drug Administration (FDA). This designation confirms that phage-based products are safe and effective tools for controlling foodborne pathogens, enhancing food safety, and protecting public health (16). Bacteriophages are considered safe and highly specific to given bacterial species, effective in killing infection-causing bacteria while leaving intact beneficial microorganisms (17, 18). Furthermore, studies on phage therapy using animal models have shown the stability of phages within different environments and conditions (18).

The pelleting process is currently the most common method of poultry feed production (19). Post-pellet liquid application (PPLA) methods were developed to solve the issue of heat-sensitive components delivery to pelleted feed. It is essentially a coating technique, in which liquid feed additives are atomized and sprayed-on feed particles, ensuring high homogeneity without disrupting the pellets. In this regard, PPLA seems to be a feasible method to incorporate bacteriophages into feed as it combines the benefits of a pelleted diet with the high bacteriophage activity. In the current study, we demonstrate that bacteriophage cocktail BAFASAL applied to poultry feed as a PPLA solution effectively reduces *Salmonella* contamination in feed.

## MATERIALS AND METHODS

### BAFASAL preparation

BAFASAL is a cocktail of four anti-*Salmonella* BPs, described in detail in Wojcik et al. 2020 (20). Cocktail is composed of four virulent phages deposited in the Polish Collection of Microorganisms under accession numbers: PCM F/00069 (strain 8sent1748), PCM F/00070 (strain 8sent65), PCM F/00071 (strain 3sent1), and PCM F/0097 (strain 5sent1). All phages show the morphology of an icosahedral head and a long non-contractile tail and are dsDNA phages, which belong to the *Caudovirales* order.

### BAFASAL production

A detailed production description was presented in Wojcik et al. 2020 (20). Briefly, in this procedure, each BP included in BAFASAL was amplified in a separate culture, and after downstream processing, was mixed at equal titer volume, giving the titer of preparation of 1 × 10^8^ PFU/mL. BAFASAL was finally tested by phage enumeration using the double agar overlay plaque assay, analyzing the microbiological sterility of the product, the endotoxins level, and the presence of bacterial genomic residues according to EFSA’s “Guidance on the characterization of microorganisms used as feed additives or as production organisms” (21).

### Infeed delivery of BAFASAL

The BAFASAL preparation was introduced into three different types of feed, the compositions of which are described in detail in Table 1.

**TABLE 1:** Composition of feed used for *Salmonella* contamination in the *in vitro* trials^*a*^

Analytical values (%)	Kokoszka Smakoszka Broiler 1, Provimi	Zielona Zagroda Layers, De Heus	Home Grown Layer 16% crumble, Purina
Crude protein	16.04	17.00	Min. 16.00
Crude fat	2.88	4.80	Min. 2.50
Crude fiber	4.21	3.60	Max. 6.00
Raw ash	13.04	4.40	
Lys	0.75	0.98	Min. 0.75
Meth	0.36	0.39	Min. 0.25
Ca	3.80	0.60	3.00–4.00
P	0.51	0.38	Min. 0.45
Na	0.16	0.16	
NaCl			0.10–0.60

^*a*^ Kokoszka Smakoszka Broiler 1, Provimi, serial No. WKA-20231220-1-0403, composed of corn, wheat, soya bean meal, wheat bran, calcium carbonate, wheat gluten feed, dried distiller’s grain (corn), sunflower seed meal from dehulled sunflower seeds, wheat grain flour, corn germ meal, rapeseed seed meal feed, sunflower vegetable oil (raw), sodium chloride, dicalcium phosphate, and milk thistle seeds. Additives per 1 kg feed: vit. A (7,000 IU), vit. D3 (2,500 IU), vit. E (10.0 mg), Fe (45.0 mg), Se (0.25 mg), Cu (5.0 mg), Mn (62.5 mg), Zn (50.0 mg), I (1.3 mg), endo-1,4-beta-xylanase (560 TXU), endo-1,4-beta-glucanase (250 TGU), 6-phytase (587 FTU), butylhydroxyanisole (0.07 mg), butylhydroxytoluene (0.96 mg), and propyl gallate (0.07 mg). Zielona Zagroda Layers, De Heus, serial No. O211310524047, composed of corn, wheat, triticale, soya extraction meal, corn meal, dried distiller’s grain, sunflower extraction meal from dehulled sunflower seeds, barley, calcium carbonate, vegetable oils and fats (sunflower, raw), rapeseed extraction meal, animal fat (pig), sodium chloride, and attapulgite. Additives per 1 kg feed: vit. A (10,000 IU), vit. D3 (3,000 IU), vit. E (30.0 mg), Fe (20.0 mg), Se (0.30 mg), Cu (15.0 mg), Mn (80.0 mg), Zn (75.0 mg), I (1.0 mg), L-lysine sulfate (3421.80 mg), clinoptilite of sedimentary origin (66.0 mg), endo-1,4-beta-xylanase (2376 U), 6-phytase (990 FTU), and salinomycin-sodium salt (60.0 mg). Home Grown Layer 16% crumble, Purina, serial No. 3004806-306, composed of processed grain by-products, grain products, plant protein products, calcium carbonate, roughage products, L-Lysine, choline chloride, pyridoxine hydrochloride, menadione sodium bisulfite complex (vit. K), manganous oxide, vit. D3 supplement, calcium pantothenate, zinc oxide, vit. E supplement, biotin, riboflavin supplement, niacin supplement, thiamine supplement, thiamine mononitrate, vit. B12 supplement, copper sulfate, vit. A supplement, folic acid, dried *Aspergillus oryzae* fermentation extract, calcium iodate, and sodium selenite.

The in-feed delivery of BAFASAL was performed in two ways.

#### Feed coating with airbrush

Two types of feed (Provimi, Kokoszka Smakoszka Broiler 1 and De Heus, Zielona Zagroda Layers) with two doses of BAFASAL were prepared for this experiment. BAFASAL was diluted 100× and 20× in sterile water to obtain the phage titers of 1 × 10^4^ PFU/g and 5 × 10^4^ PFU/g, respectively. All samples of feed were sprayed on at 1% inclusion rate.

The bottles with feed were covered with caps containing a septum, in which the tip of the airbrush was placed. The bottles were rotated around the horizontal axis during airbrush spraying. Two-bar pressure was applied during the process. At the end of spraying, the bottles were covered with regular caps, and the content was vigorously shaken. Finally, each sample was divided into proper portions.

#### Feed coating with ribbon mixer

One type of feed (Purina, Home Grown Layer 16% crumble) with 1 dose of BAFASAL was prepared for this experiment.

BAFASAL was diluted 100× in sterile water to obtain the phage titer of 1 × 10^4^ PFU/g. The feed sample was sprayed on at 1% inclusion rate.

Diluted BAFASAL was added to the reservoir, pumped by the compressor supplying the air and atomized/sprayed through the nozzles directly on feed. Once the product has completely left the reservoir, the compressor is switched off, and the mixing of the chamber contents continues for 5 min. Finally, the sample was divided into proper portions. For this process, efficiency was assessed from the ratio of the bacteriophage particles present in the feed after PPLA to the amount of bacteriophages applied on it. Log_10_ values of bacteriophage titers were used for the calculations.

### Stability and homogeneity assessment for BAFASAL in feed

The phage content of BAFASAL in feed was evaluated directly after spray-on (timepoint 0) and after 3 weeks of storage at 25°C. At both time points, BAFASAL level was determined in at least three independent feed samples.

To confirm the uniform distribution of BPs in the tested material (feed homogeneity), independent samples were collected, each from a different spot in the container.

In the case of the airbrush coating, five separate samples for each type of feed and each dose were collected directly after the process, whereas in the case of coating in the ribbon mixer, six independent samples were collected directly after the process. Then, independent dilution series were prepared for each sample and plated for bacteriophage enumeration.

In all tests, the titer of BPs was determined by the double agar overlay plaque assay. The mean log values of phage titer for those samples, along with the corresponding standard deviation and CV, were calculated. The homogeneity was measured by the coefficient of variation (CV). The target CV value in this study was <10%, which is a commonly accepted value for the homogeneity of feed additives (22).

### *In vitro* evaluation of feed protection by BAFASAL

Two independent experiments were performed on two different types of feed for poultry: Provimi and De Heus (the composition details are presented in Table 1). The following group layout was applied: PC (positive control) – no phages, *Salmonella* contamination 1 × 10^2^ CFU/g; D1 (treatment dose 1) – BAFASAL dose 1 × 10^4^ PFU/g, *Salmonella* contamination 1 × 10^2^ CFU/g; D2 (treatment dose 2) – BAFASAL dose 5 × 10^4^ PFU/g, *Salmonella* contamination 1 × 10^2^ CFU/g. In the first experiment, each group consisted of two separate replicates, and in the second experiment, each group consisted of three separate replicates.

Fresh 500 g feed samples with BAFASAL in both doses, produced by airbrush coating, were prepared for each experiment. *Salmonella* contamination was performed at two time points: 24 h and 21 days after phage infeed introduction.

For *Salmonella* contamination, lyophilized cultures of *Salmonella enterica subsp. enterica* serovar Enteritidis D ATCC 13076 were obtained from the American Type Culture Collection (ATCC) and resuscitated according to ATCC recommended methods. The culture was incubated in liquid LB medium, and the cells were harvested by centrifugation. Aliquots of cell suspension in 5% (wt/vol) saccharose were prepared and sprayed on sterile, finely ground corn grains. The grains coated with *Salmonella*, initially frozen at −50°C, were lyophilized (process conditions: vacuum 0.220 mbar, set condenser temperature −90°C, duration 24 h), vacuum-sealed, and stored at 2°C–10°C. The level of bacteria in the dried inoculum was determined by serial dilutions method. The *S*. Enteritidis was plated on xylose lysine deoxycholate (XLD) agar to confirm the desired bacterial load in corn of 5 × 10^3^–1 × 10^4^ CFU/g.

BAFASAL-protected and non-treated feed was split into 24.5 g samples according to the experimental layout and stored in sterile glass jars. Each sample from groups PC, D1, and D2 was prepared in two portions, which were contaminated with 0.5 g of *Salmonella* lyophilizate. Samples from one portion were incubated at 25°C for 24 h post-phage infeed introduction, while samples from the second portion for 21 days post-phage infeed introduction; 225 mL of sterile UPB (universal pre-enrichment broth) was added to each glass jar, and the contents were incubated at 37°C for 18 h. Serial dilutions of the supernatant were plated on xylose lysine deoxycholate (XLD) agar for *Salmonella* enumeration.

### *In vivo* evaluation of feed protection by BAFASAL

The experiment was performed at the AgriSearch Hungary Ltd, Hungary; 360 1-day-old male broilers Ross 308 were used in the experiment. Birds were vaccinated against Newcastle Disease (ND) at the hatchery and on day 21, against Infectious Bronchitis (IB) at the hatchery. The study lasted for 35 days. Mash feed (starter) or pelleted feed (grower and finisher) and water were provided to birds *ad libitum*. The composition of the feed was summarized in Table 2.

**TABLE 2:** Composition of feed used in the *in vivo* trials

Feed composition and calculated analysis of basal diets			
Ingredients (%)	Starter feed serial No. 1/256/24, (0–13 days)	Grower feed serial No. 2/256/24, (14–27 days)	Finisher feed serial No. 3/256/24, (28–35 days)
Corn	46.00	50.00	54.00
Wheat	13.50	13.00	10.00
Soybeans meal 48%	30.00	26.00	24.00
Sunflower meal 37%	3.00	3.00	3.00
Sunflower oil	3.00	4.00	5.00
Monocalcium phosphate	1.00	0.65	0.60
DL Methionine 99%	0.20	0.10	0.10
L-Lysine HCl 78%	0.25	0.20	0.20
Premix^*a*^+coccidiostat	3.00	3.00	3.00
Salt	0.05	0.05	0.10
Total	100	100	100
Analytical values (%)			
Dry matter	88.41	88.38	88.35
ME broiler, MJ/kg	12.55	12.86	13.12
Crude protein	21.82	19.72	18.05
Crude fat	5.84	6.91	7.55
Crude fiber	3.82	3.67	3.65
Crude ash	6.39	5.83	5.85
Lys (Lysine)	1.32	1.19	1.07
Meth (Methionine)	0.55	0.52	0.48
Met+Cis (Methionine+ Cysteine)	0.89	0.84	0.80
P (Phosphorus)	0.77	0.68	0.65
Available *P*	0.48	0.42	0.40
Ca (Calcium)	1.01	0.93	0.88


^*a*^ Provided per kilogram of diet: Vitamin A (E 672): 10,000 IU; Vitamin D3 (E 671): 4,000 IU; Vitamin E (a-tocopherol): 35.0 mg; Vitamin K3: 3.0 mg; Vitamin B1: 2.0 mg; Vitamin B2: 5.0 mg; Vitamin B6: 4.0 mg; Vitamin B12: 11.0 µg; Nicotinic acid: 40.0 mg; Calcium pantothenate: 12.0 mg; Folic acid: 2.0 mg; Biotin: 0.18 mg; Choline chloride: 1450 mg; Cu: 8.0 mg; Fe: 50.0 mg; I: 2 mg; Mn: 70.0 mg; Se: 0.15 mg; Zn: 80.0 mg.

**Fig 1:**
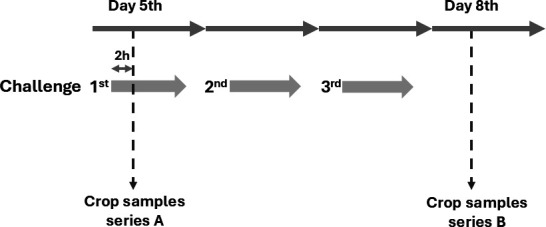
Crop sampling scheme.

Three hundred and sixty healthy 1-day-old male Ross 308 broilers were randomly allocated to 20 pens, with 18 birds per pen. Birds were distributed into four groups (five replicates per each group): NC—negative control group, PC—positive control group (birds exposed to *Salmonella*-contaminated feed), D1—treatment group (birds fed with BAFASAL-protected feed for the whole cycle in the dose of 1 × 10^4^ PFU/g and exposed to *Salmonella*-contaminated feed), and D2—treatment group (birds fed with BAFASAL-protected feed for the whole cycle in the dose of 5 × 10^4^ PFU/g and exposed to *Salmonella*-contaminated feed). All birds belonging to groups PC, D1, and D2 were exposed to *Salmonella*-contaminated feed on 3 consecutive days: day 5, day 6, and day 7. The targeted bacterial strain was *Salmonella enterica* serovar Enteritidis 12 from the culture collection of Proteon Pharmaceuticals S.A., described elsewhere as representative for at least 55 *S*. Enteritidis strains isolated across the world (6 isolates from Brazil, 1 from Egypt, 1 from the Netherlands, 42 from Poland, 1 from Spain, 2 from Turkey, and 2 from Ukraine). Birds belonging to groups PC, D1, and D2 were exposed to *Salmonella*-contaminated feed at a dose of about 1 × 10^9^ CFU/bird, previously chosen via an *in vivo* infection model study. During the trial, the following parameters were recorded: body weight at the 35th day; average daily weight gain, average daily feed intake, feed conversion ratio (FCR), and European production efficiency factor (EPEF) in the period 0–35 days. Additionally, daily health records, illness, culls, and mortality, including the reason for culls and probable cause of mortality of chickens, were recorded. On the 5th and 8th days, two birds/pen from group PC, D1, and D2 were sacrificed, and crops (outpockets of the lower esophagus) were aseptically extracted, as presented in Fig. 1 and analyzed for *Salmonella* enumeration. On days 18 and 35, two birds/pen were sacrificed, and caeca were aseptically extracted for *Salmonella* testing.

To exclude an external microbiological contamination, feed, meconium samples, and boot swabs were tested for *Salmonella* spp. presence on day 0. The presence of *Salmonella* spp. was assessed according to PN-EN ISO 6579-1:2017-04, Microbiology of food and animal feeding stuffs, Horizontal method for the detection of *Salmonella* spp. Additionally, caeca samples from NC (days 18 and 35) were also checked for *Salmonella* spp. presence. For this purpose, buffered peptone water (BPW) was added in a volume equal to 450 mL to each pair of boot swabs and each meconium sample, while for feed and cecum samples, BPW was added at a volume equal to nine times the weight of the sample. All samples were homogenized for 1–2 min (boot swabs, meconium, feed 1 min, and caeca 2 min). Samples were incubated in BPW at 37°C ± 1°C for 18 h ± 2 h. Rappaport-Vassiliadis medium with agar and novobiocin (MSRV) was inoculated with the obtained culture and incubated at 41.5°C ± 1°C for 24 h ± 3 h. If no growth of any bacteria was observed, incubation was continued for an additional 24 h ± 3 h. Xylose lysine deoxycholate agar (XLD) and Brilliant green agar (BGA) were inoculated with the obtained culture and incubated at 37°C ± 1°C for 24 h ± 3 h. After incubation, plates were examined for the growth of *Salmonella* colonies.

The number of *Salmonella* spp. was tested in crop (days 5 and 8) and caeca (days 18 and 35) from PC, D1, and D2 treatments by a miniaturized Most Probable Number (MPN) technique according to ISO/TS 6579-2:Microbiology of food and animal feed - Horizontal method for the detection, enumeration, and serotyping of *Salmonella*. Part 2: Enumeration by a miniaturized most probable number technique. BPW was added to each sample in volume equal to nine times the weight of sample and homogenized for 2 min. Afterward, 2.5 mL of previously homogenized samples were transferred in triplicate into 24-well plates. A volume of 0.5 mL of each sample was transferred into subsequent wells containing 2 mL of BPW, giving a 5-fold dilution, and mixed. This step was repeated 11 times. Plates were incubated at 37°C for 18 h ± 2 h. Subsequently, 20 µL of BPW culture was transferred to the margin of the corresponding well, onto the surface of the Rappaport-Vassiliadis medium with agar and novobiocin (MSRV) and incubated at 41.5°C for 48 h ± 3 h. Suspected cultures from MSRV wells were subcultured onto xylose lysine deoxycholate agar (XLD) and incubated at 37°C for 24 h ± 3 h.

Isolates identified on XLD or BGA as *Salmonella*-positive were confirmed according to Microbiology of food and animal feeding stuffs, Polymerase chain reaction (PCR) for the detection of food-borne pathogens:

- General requirements and definitions (PN-EN ISO 22174: 2006).
- Requirements for sample preparation for qualitative detection (PN-EN ISO 20837: 2007).
- Requirements for amplification and detection for qualitative methods (PN-EN ISO 20838: 2007).

The number of *Salmonella*-positive cultures in each series during enumeration of *Salmonella* was recorded, and the MPN value was calculated using the MPN calculation program.

### Statistical analysis

The statistical tests were adjusted to the conducted studies and analyzed with the program STATISTICA (version 14.0.1.25, TIBCO Software Inc.) and Prism 10 (version 10.0.3). For the *in vitro* evaluation of feed protection by BAFASAL, one-way ANOVA, followed by Tukey’s post hoc test was used. In the *in vivo* study, birds were allocated to treatments randomly, with the pen as the experimental unit, and for each treatment group, there were five replicates. The one-way ANOVA, followed by Tukey’s post hoc test, was applied to assess the statistical significance of the results of body weight, average daily gain, and average daily feed intake, feed conversion ratio, European production efficiency factor, and mortality. To assess the statistical significance of the obtained differences in *Salmonella* number in caeca samples, one-way ANOVA followed by Tukey’s post hoc test was performed. MPN values were log-transformed. If the result was 0, it was replaced with half the value of the lowest positive result (the lowest positive value was 0.16 MPN/g, so that negative results were replaced by 0.08 MPN/g). To assess the statistical significance of crop samples results, χ^2^ test was used. Although in crop (days 5 and 8), the number of *Salmonella* spp. was tested by a miniaturized Most Probable Number (MPN), finally, the MPN results were translated into positive/negative results due to observations that no *Salmonella* was detected in D1 and D2 at day 8. In performed statistical analyses, significant differences were declared at *P* ≤ 0.05, while 0.05 < *P* ≤ 0.10 was considered a near-significant trend.

## RESULTS

### Efficiency and homogeneity of BAFASAL delivery to feed through PPLA

The PPLA process was used to obtain BAFASAL-protected feed. On a laboratory scale, BAFASAL was applied by spraying on pelleted feed with the usage of an airbrush, while in an approach imitating the industrial process, BAFASAL was introduced into the feed through nozzles placed in the lid of a laboratory ribbon mixer. The used ribbon mixer is directly scalable into equipment for industrial-scale PPLA. The efficiency of the PPLA process in the ribbon mixer was established at a value of 97%, whereas in the laboratory process, it varied in the range of 82%–90%.

After coating the feed with BAFASAL, the homogeneity and stability of bacteriophages were checked. The mean log values of phage titer for samples tested from each batch, along with the corresponding standard deviation and CV, allowing for homogeneity evaluation, are presented in Table 3. The obtained results confirmed the uniform distribution of BAFASAL bacteriophages in feed from the ribbon mixer. In the case of the samples coated with the use of an airbrush, the phage homogeneity was lower; however, it was still at a level acceptable for the purpose of stability analyses.

**TABLE 3:** Homogeneity of bacteriophages in BAFASAL-protected feed

	Method	Feed type	PFU/g mean log_10_ value	SD	CV
BAFASAL dose 1 × 10^4^ PFU/g	Ribbon mixer	Purina	4.62	0.12	2.51%
	Airbrush	Provimi	3.91	0.15	3.84%
		De Heus	4.18	0.50	11.88%
BAFASAL dose 5 × 10^4^ PFU/g	Airbrush	Provimi	4.30	0.26	6.04%
		De Heus	4.25	0.32	7.58%

The results presented in Table 4 confirmed the 21-day stability of BAFASAL bacteriophages in different feeds for both spray-on techniques, the airbrush, and the ribbon mixer. Bacteriophage products were considered stable when over 80% of active particles was retained in the analyzed material.

**TABLE 4:** Stability of bacteriophages in BAFASAL-protected feed

	Method	Feed type	Repetition	PFU/g log_10_ value (initial titer)	PFU/g log_10_ value (21-day titer)	Stability (%)
BAFASAL dose 1 × 10^4^ PFU/g	Ribbon mixer	Purina	1	4.12	3.72	90%
		Purina	2	4.81	4.76	99%
	Airbrush	Provimi	1	4.07	3.43	84%
		Provimi	2	3.80	3.13	82%
		De Heus	1	4.71	3.71	79%
		De Heus	2	3.83	3.32	87%
BAFASAL dose 5 × 10^4^ PFU/g	Airbrush	Provimi	1	4.02	4.02	100%
		Provimi	2	4.49	3.79	84%
		De Heus	1	3.90	4.23	108%
		De Heus	2	4.48	3.70	83%

### *In vitro* evaluation of feed protection by BAFASAL

**Fig 2:**
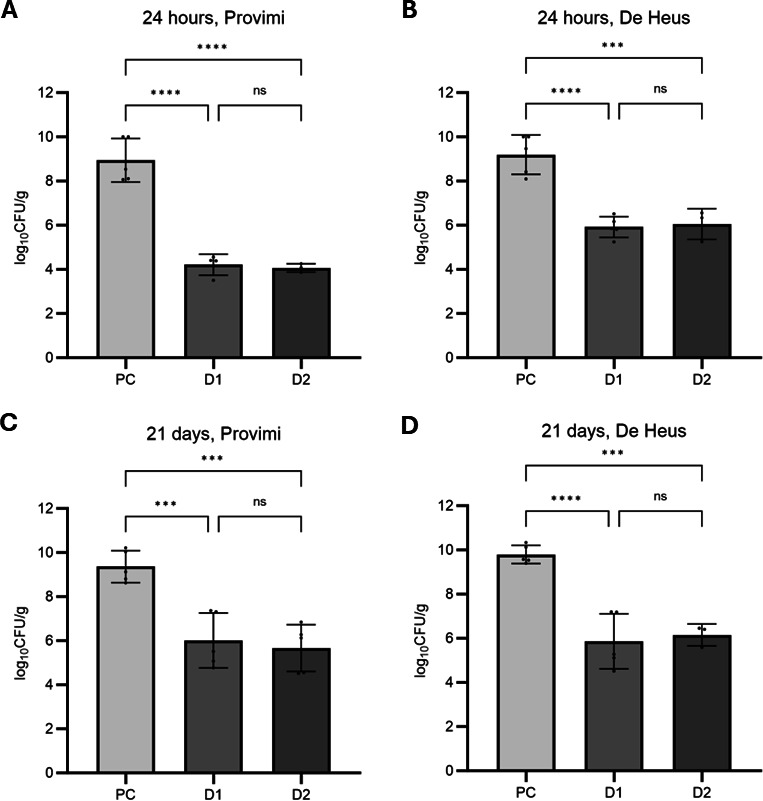
*Salmonella* reduction in feed. (**A**) Provimi feed contaminated with *Salmonella* after 24 hours of storage; (**B**) De Heus feed contaminated with *Salmonella* after 24 hours of storage; (**C**) Provimi feed contaminated with *Salmonella* after 21 days of storage; (**D**) De Heus feed contaminated with *Salmonella* after 21 days of storage. PC - positive control; D1 - BAFASAL-protected feed, dose 1 × 10^4^ PFU/g; D2 - BAFASAL-protected feed, dose 5 × 10^4^ PFU/g. Statistically significant level: ns *P* > 0.05; ***P ≤ 0.001; ****P ≤ 0.0001).

The results of the evaluation of feed protection by BAFASAL are presented in Fig. 2. It has been observed that in both tested types of feed sprayed with 1 × 10^4^ PFU/g (D1) and 5 × 10^4^ PFU/g (D2) of BPs and later artificially contaminated with bacteria, *Salmonella* levels were significantly lower when compared to feed without BPs (positive control-PC). This effect of BPs was observed both after 24 h and after long-term storage of the feed. Thus, after 24 h, the level of *Salmonella* spp. in Provimi feed (Fig. 2A) protected with low (D1) and high (D2) dose of BPs was reduced by approximately five logs compared to control (*P* < 0.001) and in De Heus feed (Fig. 2B) protected by low (D1) and high dose (D2) of BPs level of *Salmonella* spp. was reduced by approximately three logs compared to control (*P* <0.001). After long-term storage, the level of *Salmonella* spp. in Provimi feed (Fig. 2C) was reduced by over three logs in feed protected with low dose (D1) and almost four logs in feed protected with high dose (D2) of BPs compared with control (*P* < 0.001), and in De Heus feed (Fig. 2D), the level of *Salmonella* spp. protected by low (D1) and high (D2) doses was reduced by approximately four logs compared to the control (*P* <0.001).

### *In vivo* evaluation of feed protection by BAFASAL

The results presented in Table 5 showed that in crop samples obtained 2 h after first feeding with *Salmonella*-contaminated feed (Samples from series A, day 5), there was no difference between birds fed with BPs protected feed and control. *Salmonella* spp. was detected in all tested samples, proving an effective contamination through feed. In contrast, in crop samples obtained after 3 days of continuous feeding birds with *Salmonella*-contaminated feed (Samples from series B, day 8) in both groups fed with BPs protected feed (D1 and D2), there was no detectable *Salmonella* (0 out of 20 samples), while in control (group PC), 7 out of 10 samples were *Salmonella* positive. This difference in frequency of *Salmonella* positive samples was statistically significant (*P* < 0.001).

**TABLE 5:** *Salmonella* spp. presence in crop^*a*^

	*In vivo* experimental groups			*P* value
	PC	D1	D2	
Series of samples	A	A	A	
Number of positive samples (*Salmonella* spp.)	10	10	10	>0.05
Number of negative samples (no *Salmonella* spp.)	0	0	0	
Series of samples	B	B	B	
Number of positive samples (*Salmonella* spp.)	7	0	0	<0.001
Number of negative samples (no *Salmonella* spp.)	3	10	10	

^*a*^ Samples collected from 10 chickens per group, 2 chickens per replicate. PC: positive control; D1: treatment group, birds fed with BAFASAL-protected feed, dose 1 × 10^4^ PFU/g; D2: treatment group, birds fed with BAFASAL-protected feed, dose 5 × 10^4^ PFU/g. Statistically significant: *P* < 0.05.

**Fig 3:**
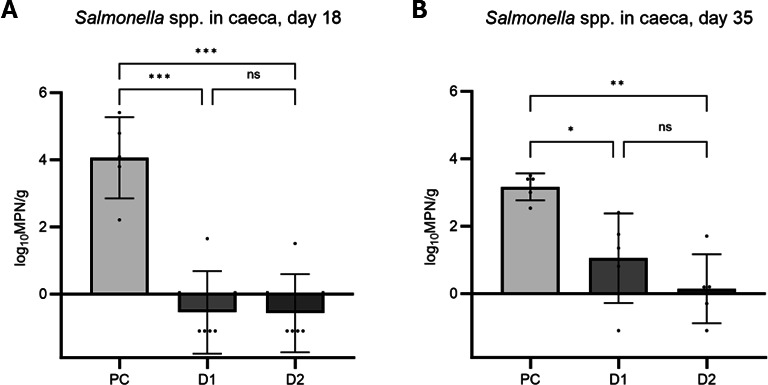
*Salmonella* spp. level in the caeca. (**A**) Day 18; (**B**) Day 35; PC - positive control; D1 - treatment group, birds fed with BAFASAL-protected feed, dose 1 × 10^4^ PFU/g; D2 - treatment group, birds fed with BAFASAL-protected feed, dose 5 × 10^4^ PFU/g. Statistically significant level: ns *P* > 0.05; *P ≤ 0.05; **P ≤ 0.01; ***P ≤ 0.001).

The results from testing the caeca content of birds culled on 18 and 35 days showed a significant reduction in *Salmonella* Enteritidis colonization of tested broilers. No *Salmonella* spp. was detected in the negative control group on both 18 and 35 days nor the samples collected for checking the background microbiological situation. On day 18, the level of *Salmonella* spp. in caeca of D1 and D2 groups was significantly reduced by over 4 logs in comparison to the positive control (*P* < 0.001). On day 35, the level of *Salmonella* in caeca in the positive control group (PC) was statistically significantly higher compared to the groups D1 (*P* < 0.05) and D2 (*P* < 0.01). The results are presented in Fig. 3.

Additionally, performance parameters (body weight, average daily weight gain, average daily feed intake, feed conversion ratio, and European production efficiency factor) assessed after 35 days of trial showed that feeding the birds with BAFASAL-protected feed resulted in the significant improvement of performance parameters. BAFASAL improved body weight, average daily gain, and average daily feed intake, showing statistically significant differences in comparison to the positive control group. Average daily feed intake in group D1 was similar to that observed for the negative control group. The feed conversion ratio was significantly higher in the positive control group compared to group D2, while in the D2 group, the result was similar to that observed for the negative control group. The main production indicator EPEF was significantly improved in groups D1 and D2 compared to the positive control group, while in the D2 group, the parameter was similar to that observed for the negative control. The mortality was monitored throughout the whole cycle, and the mortality rate evaluated at the end of the trial was significantly higher in the positive control PC compared to D1 (*P* < 0.05), while between PC and NC, D2 a near-significant trend was observed. The results are presented in Table 6.

**TABLE 6:** Assessment of performance parameters of broilers^*a*^

	*In vivo* experimental groups				*P*-value
	NC	PC	D1	D2	
BW, g	2,164 ± 30^a^	1,805 ± 60^d^	1,905 ± 46^c^	2,008 ± 47^b^	<0.001
ADG, g/bird/day	54.06 ± 0.74^a^	44.15 ± 2.3^c^	48.10 ± 1.01^b^	50.10 ± 0.61^b^	<0.001
ADFI, g/bird/day	85.75 ± 1.17^a^	78.96 ± 0.84^c^	84.60 ± 1.42^ab^	83.46 ± 1.07^b^	<0.001
FCR, kg/kg	1.59 ± 0.04^c^	1.79 ± 0.09^a^	1.76 ± 0.06 ^ab,x^	1.67 ± 0.03 ^bc,y^	<0.001
EPEF	334.30 ± 22.55 ^a,x^	223.91 ± 38.71^c^	273.75 ± 14.19^b^	294.79 ± 15.71 ^ab,y^	<0.001
Mortality, %	2.00 ± 4.47 ^ab,y^	10.00 ± 7.07 ^a,x^	0.00 ± 0^b^	2.00 ± 4.47 ^ab,y^	<0.05

^*a*^ BW: body weight, day 35; ADG: Average daily gain 0–35 days; ADFI: Average daily feed intake 0–35 days; FCR: Feed conversion ratio 0–35 days; EPEF: European Production Efficiency Factor 0–35 days. Values are presented as the mean ± SD. Average daily gain, feed intake, and feed conversion ratio data were corrected for mortality. NC: negative control; PC: positive control; D1: treatment group, birds fed with BAFASAL-protected feed, dose 1 × 10^4^ PFU/g; D2: treatment group, birds fed with BAFASAL-protected feed, dose 5 × 10^4^ PFU/g. Different superscripts denote statistically significant differences or trends (a/b/c/d: *P* ≤ 0.05; x/y: 0.05 < *P* ≤ 0.1).

## DISCUSSION

Ensuring feed safety continues to be a priority in animal production. This requires elucidating and utilizing interventions for controlling pathogens such as *Salmonella*, for which bacteriophages seem to be a solution. The anti-*Salmonella* activity of BPs has been confirmed in numerous studies, indicating their high effectiveness in combating these bacteria (6, 16, 20, 23). Bacteriophages were tested not only *in vitro* (6, 18) but also for use in protection against *Salmonella* colonization in poultry (2, 24). However, in most studies, BPs were administered directly to the animal or in drinking water (25). There are several reports indicating the effectiveness of phages in controlling *Salmonella* when added into feed, but the methods of introducing phages into feed presented there do not allow their use in large-scale production (23, 24, 26, 27). Therefore, in this work, we undertook to develop a process for introducing phages into feed, which has a large-scale application potential in the poultry industry. Delivering phages to feed creates technological challenges as BPs are sensitive to a variety of physical and chemical factors, which cause the loss of their biological functionality (27). In current animal feed production, heat treatments like extrusion or pelletization are routinely used. These processes involve moisture, high temperature, and pressure, which help with improving the digestibility of feed, but at the same time, negatively influence heat-sensitive additives (28). High sensitivity of BPs is a challenge when introducing them during the manufacturing process; simultaneously, their introduction needs to meet specific requirements of feed products, which cannot be compromised by their incorporation. Preservation of activity of thermolabile additives in feed requires application of dedicated processes such as PPLA (28). This study was conducted to demonstrate the PPLA of the BAFASAL preparation as a solution for feed preservation, leading to a reduction of *Salmonella* Enteritidis contamination in the crop and, consequently, the prevention of cecal colonization in broilers. The results confirm the potential of BAFASAL to effectively reduce *Salmonella* contamination in poultry feed and protect chickens from *Salmonella* colonization. These findings open the possibilities for the usage of BAFASAL preparation, which was previously proved to be safe not only for chicken but also for humans (20, 29).

The composition of BAFASAL reflects the general assumption that using a cocktail of bacteriophages rather than a single phage strain is limiting the emergence of bacteriophage-insensitive mutants (BIM); however, the detailed method of selection of particular cocktail components is not in the scope of the current research. This approach is particularly important in the context of combating bacteria that, when exposed to environmental stressors, have evolved diverse defense and adaptation mechanisms to enhance survival in challenging conditions. In recent years, the widespread and intensive use of antibiotics has contributed significantly to the global issue of antimicrobial resistance (AMR), affecting both human and veterinary medicine. Phage therapy presents a promising alternative to mitigate AMR. Although both antibiotics and bacteriophages can induce bacterial resistance, phages possess a distinct advantage: their ability to adapt to bacterial changes and often regain infectivity (30). Notably, the rate at which bacteria develop resistance to phages is approximately 10 times slower than the rate at which they acquire resistance to antibiotics (31). This process can be further decelerated through the use of phage cocktails—combinations of multiple phages targeting different bacterial receptors—or by integrating phages with other antimicrobial agents. Such strategies reduce the likelihood of bacterial adaptation to all selective pressures simultaneously (30). Several well-characterized bacterial defense mechanisms against bacteriophages exist. For example, bacteria can modify their surface receptors or produce outer membrane carriers to block phage attachment. In response, phages may evolve mutations in their receptor-binding proteins (RBPs), enabling them to recognize multiple host receptors. Additionally, bacteria utilize restriction-modification (R-M) systems, which involve methylation of their own DNA and cleavage of foreign, unmethylated DNA, such as that of phages. Phages counter this by modifying or deleting recognition sites in their genomes (30, 32, 33). More sophisticated defense systems include CRISPR-Cas (clustered regularly interspaced short palindromic repeats), which allows bacteria to incorporate fragments of phage DNA into their genomes to recognize and target future phage infections. In turn, phages can evade CRISPR-Cas interference through single-point mutations or by producing anti-CRISPR (Acr) proteins that disrupt the host’s defense system (34, 35). Although repeated exposure to specific phages can lead to the emergence of BIMs, this resistance often imposes a biological cost on the bacterial host, resulting in slower growth, decreased virulence, and increased susceptibility to other antimicrobial agents (36, 37). In our studies, no secondary resistance to BAFASAL was observed, supporting the product’s long-term efficacy and safety profile.

BAFASAL is the first commercial bacteriophage preparation with potential for the PPLA process. There are several commercial products that show high anti-*Salmonella* activity, but are designed for pre- and post-slaughter stages of the food chain, rather than for PPLA. SalmoFresh by Intralytix Inc. (USA) has already been approved as Generally Recognized as Safe (GRAS) by the FDA for food treatment (GRN No. 435), GPI Biotech VAM-S (GRN No. 917) by Gum Products International, Inc. (Newmarket, Canada) for use on poultry, red meat, eggs, fruits, vegetables, fish, and shellfish, SalmoPro by Phagelux (Canada) for use as an antimicrobial processing aid to control *Salmonella* on food (GRN No. 603), while Salmonelex (GRN No. 468) by Micreos Food Safety BV (The Netherlands) for use as an antimicrobial on foodstuffs to control *Salmonella*, which can be sprayed topically or added to chill tank water (2).

Delivery of BAFASAL into the feed through PPLA, in an approach imitating the industrial process with a ribbon mixer, proved to be highly efficient (97% efficiency), with good distribution of BPs (CV = 2.51%) throughout the entire volume of the feed treated with the preparation. Additionally, the high stability of the preparation in feed has been confirmed (over 80% of active BPs after 21 days), which indicates the possibility of using this process in market conditions. Our findings showed the proper matching of the selected process of infeed delivery for BAFASAL preparation. It is worth mentioning that Thanki et al. (26), who investigated the delivery of BPs powder into feed, could not maintain laboratory phage yields during scale-up in a commercial spray dryer (titers dropped by about 2 logs). However, their spray-dried phages survived mixing and pelleting in a commercial feed mill and sustained no further loss in titer when stored at 4°C or barn conditions over 6 months. This is in line with our observations on the stability of phages after introducing them to feed.

BAFASAL delivery to the feed via PPLA resulted in a feed protected from *Salmonella* contamination upon feed hydration, giving the reduction of *Salmonella* spp. by at least three logs when exposing BAFASAL-protected feed to *Salmonella*. This effect might be difficult to compare with other studies, as we were not able to identify published research on BPs efficacy in feed protection from *Salmonella* contamination, although there is some research with in-feed BPs application (24, 38, 39). It is important to consider that, as discussed by Dhowlaghar and Denes (40), bacteriophages from the feed sample can interfere with the enrichment step of *Salmonella* culture. However, in the experimental design presented in this study, bacteriophages and lyophilized *Salmonella* were introduced into the feed matrix of a low moisture content (up to 13%). This prevents bacteria and bacteriophage replication. Therefore, the actual antibacterial effect of the presence of bacteriophages will occur only upon matrix hydration. In the case of the current study, hydration occurred in the enrichment step of the *Salmonella* enumeration protocol and resulted in bacteriophage-mediated decrease in the number of bacteria. Thus, in this study, an enrichment incubation step was employed to observe the protective effect of bacteriophages. It is important to consider that resultant observations are not showing that spraying pelleted feed with bacteriophages decreases the number of *Salmonella* in dry feed. That is simply impossible because of the biological mechanism of bacteriophage activity. The conclusion we propose is that phages introduced into the feed by spraying act as a protective barrier, preventing the release and subsequent colonization of *Salmonella* in the animal’s digestive tract. The results of our animal experiments confirmed that our *in vitro* approach was appropriate and effective in this context. In summary, our results showed that the PPLA delivery of BAFASAL significantly limits the risk of feed contamination by *Salmonella*.

What is even more interesting, the results of the *in vivo* study showed that administration of BAFASAL to feed significantly reduced the *Salmonella* content in the crop of birds exposed to *Salmonella* contaminated feed within 3 days from the exposure in comparison to the group not receiving BPs. This is consistent with the previous study conducted by Gonçalves et al., who found that the use of BPs reduced the number of *Salmonella* Enteritidis in the crops of chicken broilers after BPs were delivered in drinking water (41). The effect of BPs in crops was not observed in samples obtained 2 h after challenge with *Salmonella*-contaminated feed. This might be explained by the fact that BPs are active only upon their hydration and the hydration of targeted bacteria. Available reports show that moistening of feed in crop is a gradual process with a 50% increase in feed hydration observed by 60 min (42, 43). Thus, in this time of sampling of the crop, bacteriophage activity was most likely still absent. Observation of elimination by BPs of *Salmonella* in digesta present in crop samples is consistent with observation of significant reduction of *Salmonella* content in ceca of birds exposed to *Salmonella*-contaminated feed protected by BPs compared to control. Taken together, these observations highlight bacteriophages as a promising tool to control the microbial quality of feed, which significantly decreases *Salmonella* colonization in chicken and increases the food safety for humans. Our results are consistent with previous findings. Thanki et al. demonstrated that the liquid phage cocktail added to mash diet with *ad libitum* access was effective in reducing *Salmonella* colonization in chickens (27). Similarly, Sarrami et al. showed that bacteriophages can be used as a feed additive to reduce *Salmonella* and other pathogenic bacteria in broilers, which could be a promising alternative to antibiotics (23). In another study, Nabil et al. confirmed the effectiveness of BPs in reducing *Salmonella* Typhimurium and *Salmonella* Enteritidis colonization in the cecum of broiler chickens within a short period (25). The potential of bacteriophages was also confirmed by Thanki et al., who proved that delivering phages via feed effectively reduced *Salmonella* colonization (26).

PPLA administration of BAFASAL to pelleted feed prevents *Salmonella* colonization by acting on pathogenic bacterial strains delivered with contaminated feed and protects gut microbiota, promoting intestinal balance and thereby also improving performance parameters, including body weight, average daily gain, and average daily feed intake compared to *Salmonella* challenged positive control group. These effects of BPs on broiler growth performance are consistent with previous reports (23). Thus, the combination of PPLA with a properly designed bacteriophage product is a promising way to improve feed hygiene, protecting human and animal health while reducing our reliance on chemical antimicrobials.
